# Imputation of Variants from the 1000 Genomes Project Modestly Improves Known Associations and Can Identify Low-frequency Variant - Phenotype Associations Undetected by HapMap Based Imputation

**DOI:** 10.1371/journal.pone.0064343

**Published:** 2013-05-16

**Authors:** Andrew R. Wood, John R. B. Perry, Toshiko Tanaka, Dena G. Hernandez, Hou-Feng Zheng, David Melzer, J. Raphael Gibbs, Michael A. Nalls, Michael N. Weedon, Tim D. Spector, J. Brent Richards, Stefania Bandinelli, Luigi Ferrucci, Andrew B. Singleton, Timothy M. Frayling

**Affiliations:** 1 Genetics of Complex traits, Institute of Biomedical and Clinical Sciences, Peninsula College of Medicine and Dentistry, University of Exeter, Exeter, United Kingdom; 2 Wellcome Trust Centre for Human Genetics, University of Oxford, Oxford, United Kingdom; 3 Department of Twin Research and Genetic Epidemiology, King's College London, London, United Kingdom; 4 Longitudinal Studies Section, Clinical Research Branch, Gerontology Research Center, National Institute on Aging, Baltimore, Maryland, United States of America; 5 Laboratory of Neurogenetics, National Institute of Aging, Bethesda, Maryland, United States of America; 6 Department of Molecular Neuroscience and Reta Lila Laboratories, Institute of Neurology, UCL, London, United Kingdom; 7 Departments of Medicine, Human Genetics, Epidemiology and Biostatistics, Jewish General Hospital, McGill University, Montreal, Quebec, Canada; 8 Epidemiology and Public Health, Institute of Biomedical and Clinical Sciences, Peninsula College of Medicine and Dentistry, University of Exeter, Exeter, United Kingdom; 9 Tuscany Regional Health Agency, Florence, Italy, I.O.T. and Department of Medical and Surgical Critical Care, University of Florence, Florence, Italy; 10 Geriatric Unit, Azienda Sanitaria di Firenze, Florence, Italy; Johns Hopkins University, United States of America

## Abstract

Genome-wide association (GWA) studies have been limited by the reliance on common variants present on microarrays or imputable from the HapMap Project data. More recently, the completion of the 1000 Genomes Project has provided variant and haplotype information for several million variants derived from sequencing over 1,000 individuals. To help understand the extent to which more variants (including low frequency (1% ≤ MAF <5%) and rare variants (<1%)) can enhance previously identified associations and identify novel loci, we selected 93 quantitative circulating factors where data was available from the InCHIANTI population study. These phenotypes included cytokines, binding proteins, hormones, vitamins and ions. We selected these phenotypes because many have known strong genetic associations and are potentially important to help understand disease processes. We performed a genome-wide scan for these 93 phenotypes in InCHIANTI. We identified 21 signals and 33 signals that reached *P*<5×10^−8^ based on HapMap and 1000 Genomes imputation, respectively, and 9 and 11 that reached a stricter, likely conservative, threshold of *P*<5×10^−11^ respectively. Imputation of 1000 Genomes genotype data modestly improved the strength of known associations. Of 20 associations detected at *P*<5×10^−8^ in both analyses (17 of which represent well replicated signals in the NHGRI catalogue), six were captured by the same index SNP, five were nominally more strongly associated in 1000 Genomes imputed data and one was nominally more strongly associated in HapMap imputed data. We also detected an association between a low frequency variant and phenotype that was previously missed by HapMap based imputation approaches. An association between rs112635299 and alpha-1 globulin near the *SERPINA* gene represented the known association between rs28929474 (MAF = 0.007) and alpha1-antitrypsin that predisposes to emphysema (*P* = 2.5×10^−12^). Our data provide important proof of principle that 1000 Genomes imputation will detect novel, low frequency-large effect associations.

## Introduction

Genome-wide association (GWA) studies have identified many novel associations between common genetic variants and human traits. These studies capture a large proportion of SNP-based common variation in the human genome but are less efficient at capturing low frequency and rare variants.

**Figure 1:**
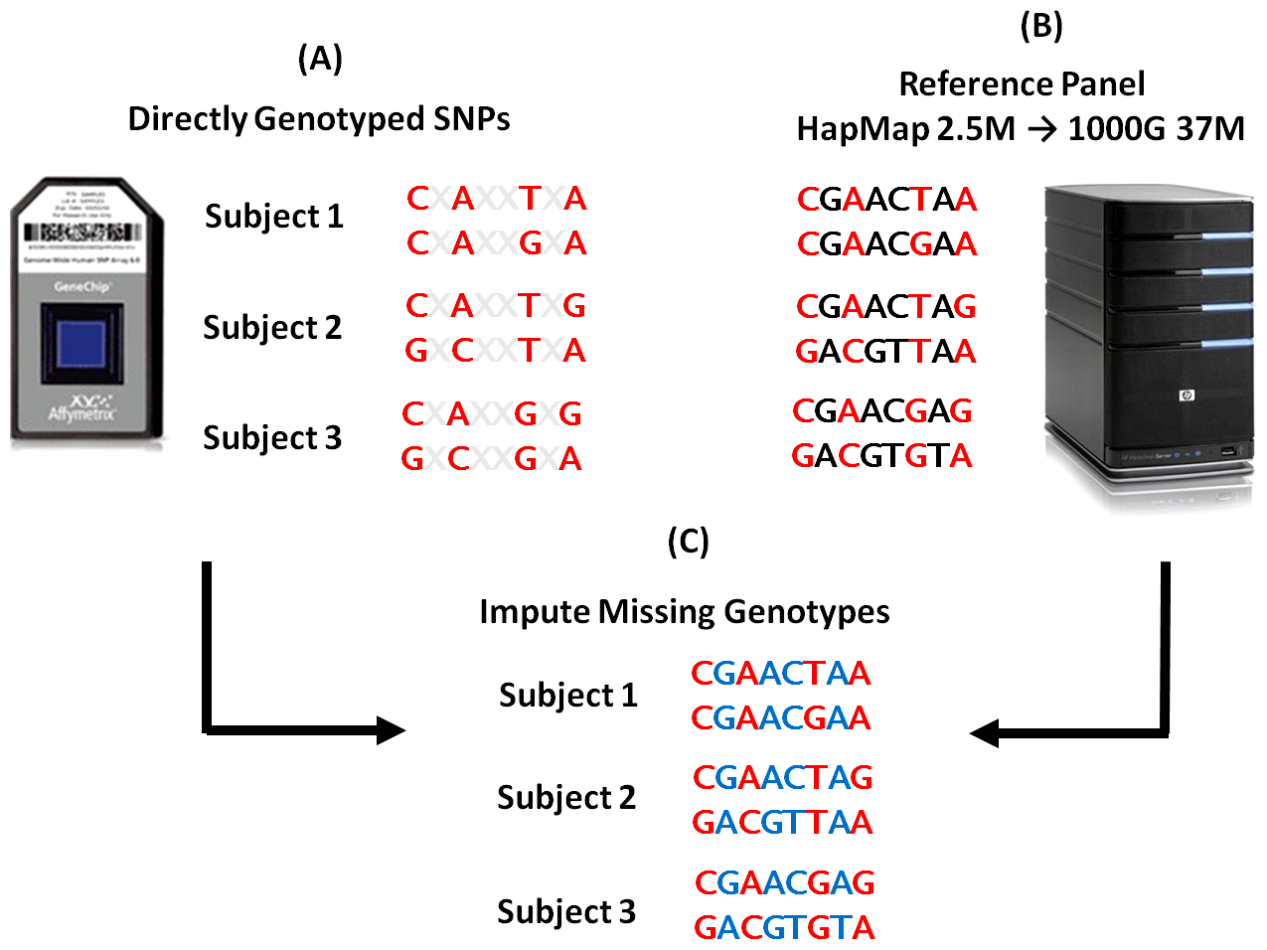
Schematic overview of imputation. (**A**) Directly genotyped SNPs are phased to estimate haplotypes that alleles reside on. (**B**) Publically available reference haplotypes from projects including the International HapMap Project and the 1000 Genomes Project are downloaded that contain variants to be imputed. (**C**) Phased haplotypes from the study are assessed and likened to publically available haplotypes. The most likely genotypes are imputed into the study based on the alleles found in reference haplotype panel.

Imputation is the process of inferring missing data based on known data [1]. Imputation is useful for performing GWA meta-analyses across studies with the same traits but different genotyping arrays. Imputation can also potentially identify signals of association not detected by direct genotypes. In the simplest scenario, a causal variant, or the most strongly associated genetic variant, may not be directly genotyped and may exist on a haplotype that is optimally captured by two rather than one directly genotyped SNP (**Figure 1**).

There are now two main sources of reference genotypes for imputation of missing variants. The International HapMap Project Phases 1 to 3 [2]–[5] consists of 4.1 million directly genotyped SNPs in 1,486 individuals and has facilitated the imputation of approximately 2.5 million SNPs in standard GWA studies. More recently, the 1000 Genomes Project has completed the low-pass sequencing of the whole genome from 1,094 individuals from a range of ethnic groups [6]. The project has annotated >37 million variants. The sequencing based approach and the large number of individuals means these variants include many low frequency (1% ≤ MAF <5%) and rare (MAF<1%) variants.

In this study we tested the hypothesis that imputation of variants identified by the 1000 Genomes Project (including low frequency and rare variants) would enhance previously identified associations and identify novel associations. We selected 93 traits measured in ∼1,200 individuals in the InCHIANTI study (**Table 1** and **Table S1**). All traits were continuous measures of circulating factors [7], [8]. These traits included circulating lipids, proteins, ions and vitamins and were selected for two reasons. First, for many traits, strong genetic associations have previously been observed in relatively small sample sizes, and so we potentially had good power to detect novel effects and stronger signals at known loci. These signals include those far exceeding genome-wide significance in our dataset alone and therefore offer a good opportunity to assess whether or not 1000 Genomes imputation can improve and refine known associations. Second, identifying genetic associations with circulating factors is potentially important to help understand disease processes. Many circulating factors are altered in disease cases compared to controls, but it is rarely understood if these factors are causal, confounded or secondary to disease processes. Genetic variants can be used to help identify causal pathways using Mendelian randomisation [9]–[12].

**Table 1:** Basic characteristics of the 1210 InCHIANTI subjects at baseline.

Characteristic	Mean (Range) or%
Age (years)	68.2 (21–102)
Sex (% male)	44.6%
BMI	27.2 (18–46.6)
Current smokers (% case)	18.8%
History of hypertension (% case)	38.4%
Treatment for hypertension - former and current (% case)	30.1%
History of diabetes (% case)	9.5%
Treatment for diabetes - former and current (% case)	6.58%
History of myocardial infarction (% case)	3.6%
Treatment for myocardial infarction - former and current (% case)	2.0%

## Results

### Variation captured by HapMap and 1000 Genomes

After applying quality control metrics we captured 2,493,682 and 10,879,115 SNPs from HapMap (release 22) and 1000 Genomes imputation (November 2010, Phase1-α), respectively (**Table S2**, **Figure S1**).

**Figure 2:**
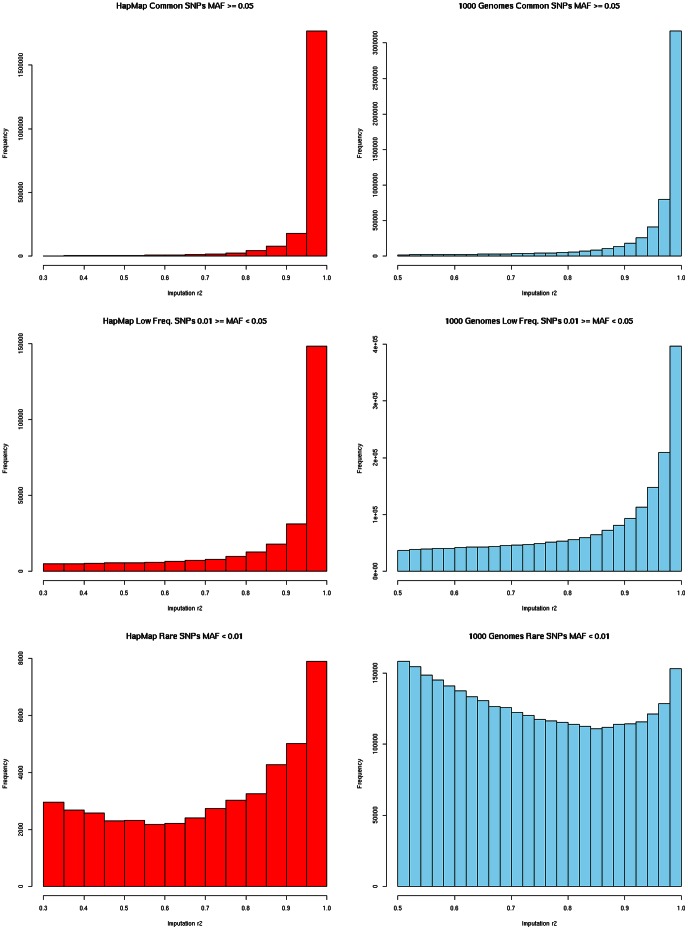
Distributions of imputation quality (*r* ^2^) across different minor allele frequency (MAF) bins. Note differences in Y axes scales.

The 1000 Genomes based imputation captured many more variants at high quality (defined as *r* ^2^_imp_>0.3 in HapMap imputation and *r* ^2^_imp_>0.5 in 1000 Genomes imputation) compared to the HapMap based imputation (**Figure 2**).

We captured 2,172,804 and 5,737,929 common SNPs (MAF≥5%) based on HapMap and 1000 Genomes based imputation, respectively. For low frequency variants (1% ≤ MAF <5%) we captured 274,995 and 1,953,185 based on HapMap and 1000 Genomes imputation respectively. For rare variants (MAF<1%) we captured 45,883 and 3,188,001 rare SNPs based on the HapMap and 1000 Genomes based imputation, respectively.

### Identification of association signals using HapMap and 1000 Genomes Imputation

#### Associations identified by HapMap imputation

Using HapMap based imputation we identified 21 signals of association at *P*<5×10^−8^ including 19 circulating factors (**Table 2**). Nine of these signals reached the stricter statistical threshold of *P*<5×10^−11^. Of these 21 signals, 14 were known, based on the same or an *r* ^2^>0.8 proxy to our index SNP reaching *P*<5×10^−8^ in the NHGRI catalogue or in other published GWA studies with the same or very similar trait.

**Table 2:** Associations of circulating factors based on HapMap imputation ordered by minor allele frequency group (<5% or ≥5), chromosome and base-pair position.

Trait	GC	Chr	SNP	BP (b37)	MAF	P*	Nearest Gene	Annotation
Alkaline phosphatase	1.055	12	rs1880889	41721235	0.03	1.96E-08	*PDZRN4*	Known
Homocysteine	1.043	1	rs1801133	11856378	0.495	1.75E-11	*MTHFR*	Known
Vitamin B6	1.017	1	rs4654748	21786068	0.499	8.84E-09	*NBPF3*	Known
Interleukin-6R	1.134	1	rs8192284	154426970	0.385	3.60E-53	*IL6R*	Known
C-reactive protein	1.035	1	rs12741825	159670145	0.301	1.08E-08	*CRP*	Known
Oxidized LDL	1.029	2	rs676210	21231524	0.239	4.05E-30	*APOB*	Known (other trait)
Macrophage inflammatory protein-1b	1.016	3	rs1500004	46150937	0.104	2.06E-08	*XCR1/CCR1*	Novel
Lipoprotein a	1.057	6	rs10455872	161010118	0.056	1.18E-20	*LPA*	Known
Insulin-like growth factor bp-3	1.038	7	rs10260816	46010100	0.456	4.42E-14	*IGFBP3*	Known
90K Protein	1.016	8	rs406652	16042507	0.159	1.56E-09	*MSR1*	Novel
Interleukin-18	1.047	11	rs2250417	112085316	0.44	2.94E-14	*BCO2*	Known
Vitamin E alpha tocopherol	1.066	11	rs964184	116648917	0.152	1.33E-08	*ZNF259*	Known
Triglycerides	1.052	11	rs964184	116648917	0.152	9.03E-09	*ZNF259*	Known
Soluble transferrin receptor	1.04	11	rs1242229	117062370	0.129	6.55E-09	*SIDT2*	Known
HDL cholesterol	1.033	16	rs1800775	56995236	0.486	8.01E-13	*CETP*	Known
Alpha-2 globulin (%)	1.058	16	rs2000999	72108093	0.228	2.13E-08	*HPR*	Known (other trait)
Lutein	1.026	16	rs11645428	81258896	0.468	1.17E-13	*PKD1L2*	Known
Beta carotene	1.051	16	rs6564851	81264597	0.364	1.70E-10	*PKD1L2/BCMO1*	Known
Free thyroxine (FT4)	1.03	17	rs7212734	6340460	0.444	1.10E-08	*AIPL1*	Novel
Macrophage inflammatory protein-1b	1.016	17	rs1015673	34477386	0.443	1.25E-22	*TBC1D3B*	Known
Vitamin E alpha tocopherol	1.066	19	rs10401969	19407718	0.057	9.32E-09	*SUGP1*	Known (other trait)

GC  =  genomic control factor used to adjust *P*-value, Chr  =  chromosome, BP (b37)  =  human genome build 37 base pair position, SNP  =  index SNP representing signal, MAF  =  minor allele frequency, P*  =  *P*-value derived after applying genomic control.

Annotations refer to whether associations represent known (present in NHGRI catalogue at *P*<5×10^−8^ or GWAS literature) signals for same trait, different traits or represent putative novel signals.

A further 3 signals were classified as known signals but with different traits. A signal near the *HPR* gene was associated with alpha-2 globulin and was previously reported as associated with haptoglobulin. A signal near the *APOB* gene was associated with oxidized LDL and was previously associated with HDL and triglycerides, and a signal near the *SUGP1* gene was associated with vitamin E and was previously associated with LDL cholesterol, total cholesterol and triglycerides.

Of the remaining 4 signals, 3 were putative novel associations (**Table 2**), and one was an association with hsCRP and represented a previously known locus – our index SNP was correlated with an *r* ^2^ of 0.32 with a published hsCRP associated variant (rs876537 [13]) ∼7 kb upstream of the *CRP* gene.

#### Associations identified by 1000 Genomes imputation

Using 1000 Genomes based imputation we identified 33 signals of association at *P*<5×10^−8^ across 30 circulating factors (**Table 3**). Eleven of these signals reached a stricter statistical threshold of *P*<5×10^−11^. These 33 signals included 14 of the 21 identified by HapMap based imputation (*r* ^2^ between Index HapMap and index 1000 Genomes SNP >0.8 of which 6 were the same lead SNP in both analyses) and 6 signals that represented the same loci as detected by HapMap based imputation (0.2<*r* ^2^<0.8 between the 1000 Genomes and HapMap index SNPs) (**Table 4**). There was one HapMap based signal of association not detected by 1000 Genomes based imputation – rs12741825 representing CRP which did not reach genome-wide significance having passed 1000 Genomes imputation quality control (*P* = 1×10^−7^, *r* ^2^_imp_ >0.99).

**Table 3:** Genome-wide significant associations of circulating factors based on 1000 Genomes imputation ordered by minor allele frequency group (<5% or ≥5), chromosome and base-pair position.

Trait	GC	Chr	SNP	BP (b37)	MAF	P**	Nearest Gene	Annotation
Total cholesterol	1.049	1	rs116435220	46890686	0.011	2.18E-08	*FAAH*	Novel
Tumor necrosis factor-a	1.024	2	rs188141385	14975369	0.004	4.16E-10	*FAM84A*	Novel
Beta globulins (%)	1.034	4	rs28645201	91528927	0.02	1.22E-09	*FAM190A*	Novel
Vitamin B12	1.055	5	rs146226203	150185826	0.012	3.01E-08	*C5ORF62/IRGM*	Novel
Lipoprotein a	1.057	6	rs55730499	161005610	0.048	3.43E-25	*LPA*	Known
Cortisol:DHEAS ratio	1.006	7	rs34670419	99130834	0.028	2.35E-08	*ZKSCAN5*	Known
Dehydroepiandrosterone	1.023	7	rs34670419	99130834	0.028	2.07E-09	*ZKSCAN5*	Known
Cl-	1.075	7	rs2371549	144172841	0.005	5.38E-09	*TPK1*	Novel
Alkaline phosphatase	1.055	12	rs1880889	41721235	0.029	3.08E-08	*PDZRN4*	Known
Alpha-1 globulin (%)	1.054	14	rs112635299	94838142	0.007	2.51E-12	*SERPINA1*	Novel
Alpha-2 globulin (%)	1.058	17	rs181929163	63740545	0.01	1.57E-08	*CEP112*	Novel
Homocysteine	1.043	1	rs1801133	11856378	0.497	2.01E-11	*MTHFR*	Known
Vitamin B6	1.017	1	rs4654932	21809436	0.417	7.39E-09	*NBPF3*	Known
Interleukin-6R	1.134	1	rs12730935	154419892	0.377	2.28E-53	*IL6R*	Known
Oxidized LDL	1.029	2	rs676210	21231524	0.239	4.12E-30	*APOB*	Known (other trait)
Macrophage inflammatory protein-1b*	1.016	3	rs113341849	46384204	0.09	2.14E-11	*CCR2*	Novel
Insulin-like growth factor bp-3	1.038	7	rs71550311	45987430	0.459	1.39E-14	*IGFBP3*	Known
90K Protein*	1.016	8	rs28491433	16050871	0.148	6.03E-10	*MSR1*	Novel
Beta Cryptoxanthin	1.021	8	rs75226183	77295977	0.087	1.69E-09	*LOC100192378*	Novel
Creatine phosphokinase	1.029	10	rs61871700	101828261	0.28	4.18E-08	*CPN1*	Known
Interleukin-18	1.047	11	rs2250417	112085316	0.44	2.97E-14	*BC02*	Known
Vitamin E alpha tocopherol	1.066	11	rs964184	116648917	0.151	6.66E-09	*ZNF259*	Known
Triglycerides	1.052	11	rs964184	116648917	0.151	1.01E-08	*ZNF259*	Known
Soluble transferrin receptor	1.04	11	rs7940310	117024481	0.13	7.17E-09	*PAFAH1B2*	Known
HDL cholesterol	1.033	16	rs711752	56996211	0.436	8.32E-13	*CETP*	Known
Alpha-2 globulin (%)	1.058	16	rs2287997	72140553	0.227	1.45E-10	*DHX38*	Known (other trait)
Beta carotene	1.051	16	rs12926540	81258987	0.362	6.98E-11	*PKD1L2/BCMO1*	Known
Lutein	1.026	16	rs9708919	81260786	0.391	7.84E-14	*PKD1L2/BCMO1*	Known
Free thyroxine (FT4)*	1.03	17	rs58926603	6341870	0.393	5.62E-09	*AIPL1/FAM64A*	Novel
Macrophage inflammatory protein-1b	1.016	17	rs4796217	34819191	0.345	6.62E-21	*TBC1D3G*	Known
Vitamin E alpha tocopherol	1.066	19	rs58542926	19379549	0.064	1.18E-08	*TM6SF2*	Known (other trait)
LDL cholesterol	1.041	19	rs1065853	45413233	0.075	1.76E-09	*APOE*	Known
Gamma-glutamyltransferase	1.043	22	rs2006227	24995756	0.386	1.70E-08	*GGT1*	Known

* =  associations that represent novel signals in both HapMap and 1000 Genomes-based analyses, P**  =  *P*-value derived after applying genomic control.

**Table 4:** The 20 signals and loci found in both HapMap and 1000 Genomes ordered by minor allele frequency group (<5% or ≥5) and chromosome.

Trait	Chr	HapMap-based Analysis			1000 Genomes-based Analysis			BP Dist.	LD (*r* ^2^)
		SNP	MAF	P*	SNP	MAF	P*		
Alkaline Phosphatase	12	rs1880889	0.03	1.96E-08	rs1880889	0.029	3.08E-08	–	–
Interleukin-6R	1	rs8192284	0.385	3.60E-53	rs12730935	0.377	2.28E-53	7078	0.957
Homocysteine	1	rs1801133	0.495	1.75E-11	rs1801133	0.497	2.01E-11	–	–
Vitamin B6	1	rs4654748	0.499	8.84E-09	rs4654932	0.417	7.39E-09	23368	0.744
Oxidized LDL	2	rs676210	0.239	4.05E-30	rs676210	0.239	4.12E-30	–	–
Macrophage inflammatory protein-1b	3	rs1500004	0.104	2.06E-08	rs113341849	0.09	2.14E-11	233267	0.667
Lipoprotein a	6	rs10455872	0.056	1.18E-20	rs55730499	0.048	3.43E-25	4508	0.888
Insulin-like growth factor bp-3	7	rs10260816	0.456	4.42E-14	rs71550311	0.459	1.39E-14	22670	0.871
90K Protein	8	rs406652	0.159	1.56E-09	rs28491433	0.148	6.03E-10	8364	0.932
Soluble transferrin receptor	11	rs1242229	0.129	6.55E-09	rs7940310	0.13	7.17E-09	37889	0.956
Triglycerides	11	rs964184	0.152	9.03E-09	rs964184	0.151	1.01E-08	–	–
Vitamin E alpha tocopherol	11	rs964184	0.152	1.33E-08	rs964184	0.151	6.66E-09	–	–
Interleukin-18	11	rs2250417	0.44	2.94E-14	rs2250417	0.44	2.97E-14	–	–
Alpha-2 globulin (%)	16	rs2000999	0.228	2.13E-08	rs2287997	0.227	1.45E-10	32460	0.937
Beta Carotene	16	rs6564851	0.364	1.70E-10	rs12926540	0.362	6.98E-11	5610	0.954
Lutein	16	rs11645428	0.468	1.17E-13	rs9708919	0.391	7.84E-14	1890	0.53
HDL cholesterol	16	rs1800775	0.486	8.01E-13	rs711752	0.436	8.32E-13	975	0.774
Macrophage inflammatory protein-1b	17	rs1015673	0.443	1.25E-22	rs4796217	0.345	6.62E-21	341805	0.577
Free thyroxine (FT4)	17	rs7212734	0.444	1.10E-08	rs58926603	0.393	5.62E-09	1410	0.547
Vitamin E alpha tocopherol	19	rs10401969	0.057	9.32E-09	rs58542926	0.064	1.18E-08	28169	1

SNP  =  index SNP representing signal, MAF  =  minor allele frequency, P*  =  *P*-value derived after applying genomic control correction, BP Dist.  =  base pair distance between the HapMap and 1000 Genomes index SNPs, LD (r^2^)  =  linkage disequilibrium measure based on *r* ^2^ correlation.

Fourteen associations represent the same signals (*r* ^2^>0.8) of which 6 are represented by the same index SNP. The remaining 6 represent the same locus (0.2<*r* ^2^<0.8).

**Figure 3:**
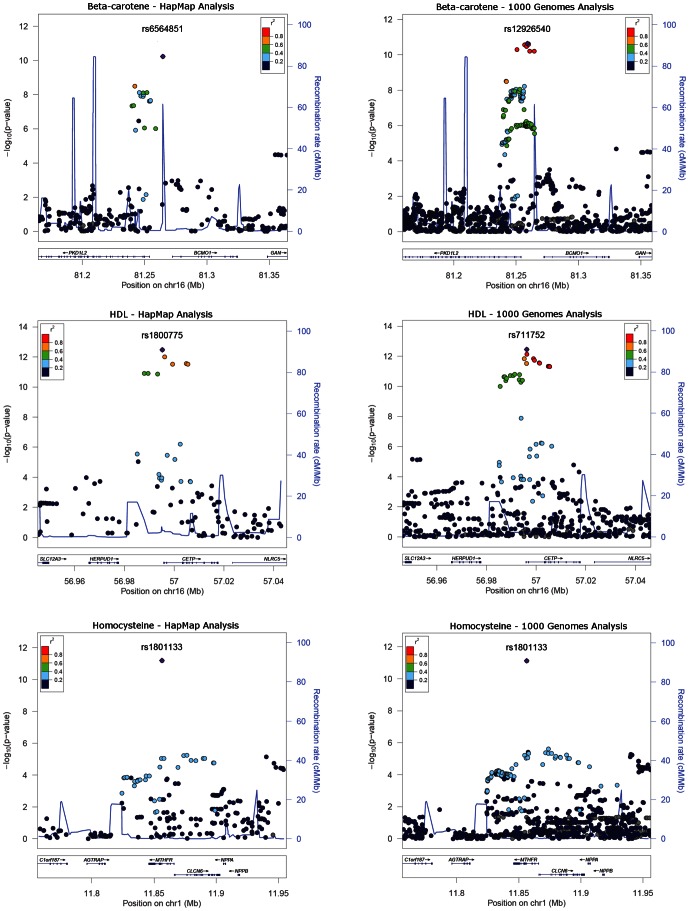
Locus zoom plots of 6 loci reaching p<5×10^−8^ in both the HapMap- and the 1000 Genomes imputed data and that represent known associations (*r* ^2^>0.2 in the NHGRI GWAS catalogue).

**Figure 4:**
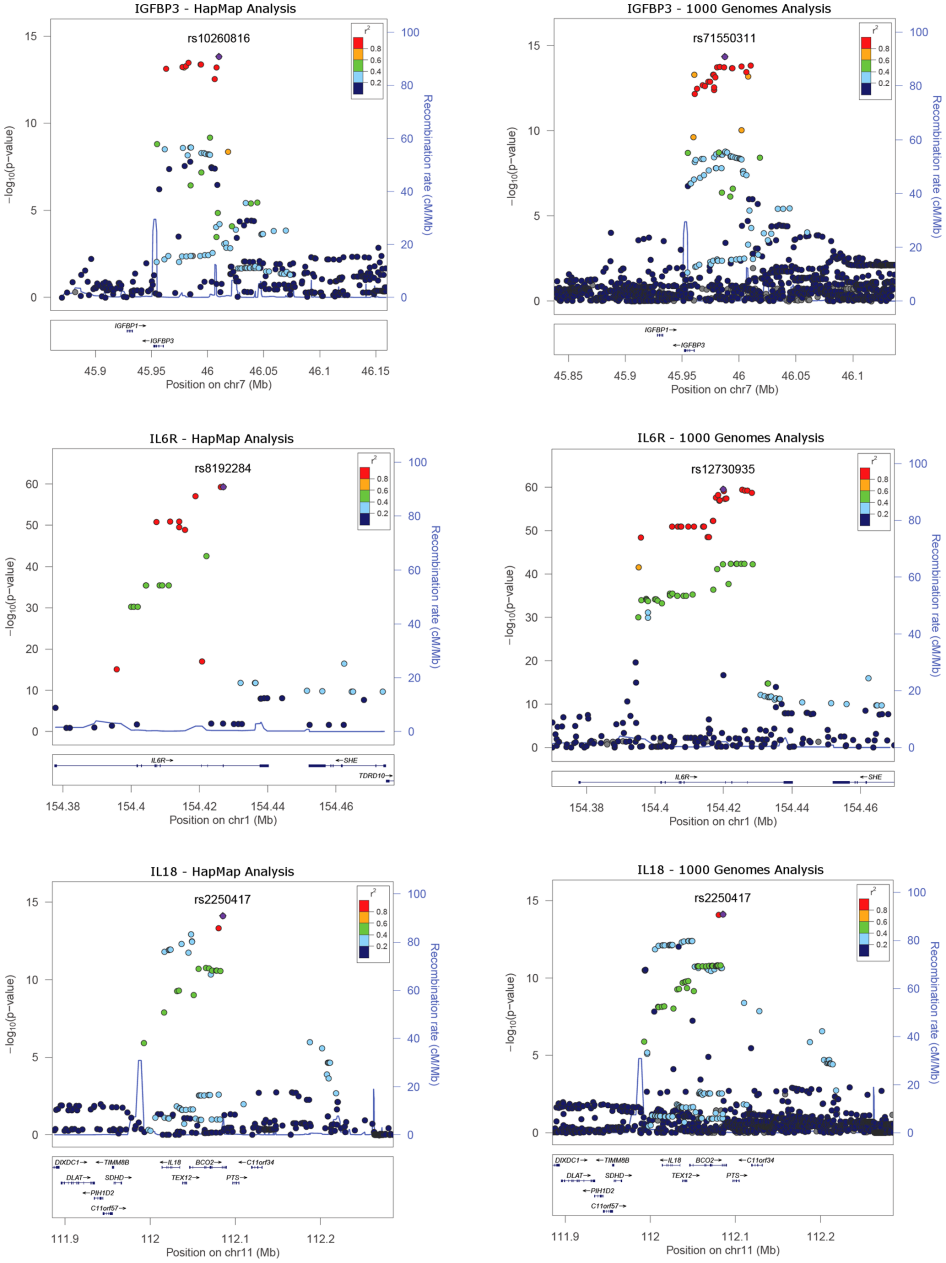
Locus zoom plots of 6 loci reaching p<5×10^−8^ in both the HapMap- and the 1000 Genomes imputed data and that represent known associations (*r* ^2^>0.2 in the NHGRI GWAS catalogue).

Of the 13 signals of association detected by 1000 Genomes imputation that were not detected by HapMap imputation (at *P*<5×10^−8^) in our study (**Table 5**), eight represented putative novel signals. The remaining five signals represented known loci (0.2<*r* ^2^<0.8 with index SNPs in NHGRI GWAS catalogue). Plots of six of the 22 associations detected at *P*<5×10^−8^ in the 1000 genomes imputation analysis and that represent previously known signals or loci (*r* ^2^>0.2 with SNPs in the NHGRI GWAS catalogue) are shown in **Figure 3** and **Figure 4**.

**Table 5:** The 13 signals identified as having an associated 1000 Genomes-based imputed SNP reaching genome-wide significance where the best HapMap SNP did not, ordered by minor allele frequency group (<5% or ≥5) and the minor allele frequency of the 1000 Genomes SNP.

Trait	HapMap-based Analysis			1000 Genomes-based Analysis				BP Dist.	LD (*r* ^2^)	Annotation
	SNP	MAF	P*	SNP	MAF	P*	Beta			
Tumor necrosis factor-a	rs10206283	0.289	5.40E-03	rs188141385	0.004	4.16E-10	−2.101	218450	0.002	Novel
Cl-	rs10262256	0.091	2.21E-02	rs2371549	0.005	5.38E-09	−2.059	508371	0.001	Novel
Alpha-1 globulin (%)	rs10149388	0.054	2.25E-04	rs112635299	0.007	2.51E-12	−1.891	371308	0.034	Novel
Alpha-2 globulin (%)	rs16959655	0.079	1.58E-03	rs181929163	0.01	1.57E-08	1.472	656607	0.004	Novel
Total cholesterol	rs17102098	0.021	1.16E-05	rs116435220	0.011	2.18E-08	−1.243	149475	0.526	Novel
Vitamin B12	rs3797617	0.146	1.58E-03	rs146226203	0.012	3.01E-08	−1.38	566327	0.009	Novel
Beta globulins (%)	rs10516879	0.163	1.51E-03	rs28645201	0.02	1.22E-09	1.116	211623	0.032	Novel
Dehydroepiandrosterone	rs11761528	0.092	9.67E-07	rs34670419	0.028	2.07E-09	−0.78	12033	0.263	Known
Cortisol:DHEAS ratio	rs10278040	0.045	1.17E-05	rs34670419	0.028	2.35E-08	0.721	10539	0.588	Known
LDL cholesterol	rs445925	0.114	3.60E-05	rs1065853	0.075	1.76E-09	−0.526	2407	0.714	Known
Beta Cryptoxanthin	rs1464093	0.178	5.05E-06	rs75226183	0.087	1.69E-09	−0.514	821919	0.049	Novel
Creatine phosphokinase	rs17112705	0.295	6.92E-07	rs61871700	0.28	4.18E-08	0.257	108933	0.78	Known
Gamma-glutamyltransferase	rs2017869	0.394	7.94E-08	rs2006227	0.386	1.70E-08	0.287	1553	0.973	Known

P* - *P*-value derived after applying genomic control correction.

Details of the best HapMap SNP within a 2 MB window centred on the 1000 Genomes SNP are provided. Annotation “Novel” refers to signals not previously reported. Annotation “known” refers to signals not identified by HapMap but identified in the NHGRI GWAS catalogue.

### Known and putative novel signals identified by 1000 Genomes that were missed by imputation with Hapmap

For the 13 signals detected by the 1000 Genomes imputation but not by the HapMap imputation analysis, we calculated linkage disequilibrium between the index 1000 Genomes SNP and the most strongly associated HapMap SNPs within a 2 Mb window centred on the 1000 Genomes SNP (**Table 5**). One of the 13 signals of association was present in the HapMap data with a proxy SNP at *r* ^2^>0.8, four with a proxy SNP at 0.5<*r* ^2^<0.8 and one with a proxy SNP at *r* ^2^ = 0.26. As expected, HapMap associations for these six signals were close to genome-wide significance (*P* = 1.2×10^−5^ to 7.9×10^−8^).

The remaining 7 signals detected in the 1000 Genomes imputation analyses but not by the HapMap based analysis appeared to represent signals specifically detected by 1000 Genomes imputation. These seven signals had very low correlations (*r* ^2^<0.05) with the most strongly associated HapMap SNP (**Table 5**). Six of these seven signals represented putative low frequency – large effect variants, with minor allele frequencies ≤ 0.02 and per allele effect sizes of 1.1 – 2.1 standard deviations (SDs). We therefore examined these loci in more detail. We checked imputed dosages and trait values for the small number of heterozygous individuals that were driving these associations, but found no obvious sources of error (**Figure S2**). All but one of these 7 signals had a proxy SNP that also showed strong evidence of association, even if the association did not reach *P*<5×10^−8^.

### Does 1000 Genomes imputation identify stronger associations at loci identified by HapMap based imputation?

We identified 14 loci associated with 13 traits where both the HapMap and 1000 Genomes index SNPs were *P*<5×10^−8^ and differed from each other (**Table 4** **and** **Table 6**). In the multivariable SNP analyses that included the two SNPs at each locus against the relevant phenotype, the 1000 Genomes SNP was more strongly associated in 13 of 14 loci (one-tail Binomial *P* = 0.0009). The *P*-value improvements were marginal for 8 of these associations and the 1000 genomes signal was not statistically stronger, but 5 of the 14 associations, showed evidence of stronger signals at *P*<0.05 and three at *P*<0.01, **Table 6**).

**Table 6:** Results from the univariable and multivariable analysis using the Index HapMap and 1000 Genomes SNPs for 13 traits across 14 loci where both index SNPs were genome-wide significant and differed from each other.

Trait	Index HM SNP	HM Uni P*	HM Multi P*	Index 1 KG SNP	1 KG Uni P*	1 KG Multi P*
90 K Protein	rs406652	2.33E-09	0.90	rs28491433	4.29E-10	0.07
Alpha-2 globulin (%)	rs2000999	2.79E-10	0.78	rs2287997	1.01E-10	0.14
Vitamin E alpha tocopherol	rs10401969	1.07E-08	0.80	rs58542926	9.38E-09	0.58
Beta carotene	rs6564851	1.26E-10	0.86	rs12926540	4.73E-11	0.1625
HDL cholesterol	rs1800775	1.98E-12	0.17	rs711752	4.77E-13	0.0305
Free thyroxine (FT4)	rs7212734	8.49E-09	0.04	rs58926603	4.39E-09	0.0342
Insulin-like growth factor bp-3	rs10260816	2.07E-14	0.39	rs71550311	6.16E-15	0.0774
Interleukin-6R	rs8192284	8.84E-60	0.19	rs12730935	4.87E-60	0.0882
Lipoprotein a	rs10455872	5.06E-25	0.44	rs55730499	2.34E-26	0.0097
Lutein	rs11645428	5.96E-14	0.01	rs9708919	4.03E-14	0.0072
Macrophage inflammatory protein-1b	rs1015673	1.37E-23	0.002	rs4796217	1.09E-21	0.2842
Macrophage inflammatory protein-1b	rs1500004	9.33E-09	0.68	rs113341849	1.37E-11	0.0003
Soluble transferrin receptor	rs1242229	7.51E-09	0.71	rs7940310	5.66E-09	0.4052
Vitamin B6	rs4654748	6.73E-09	0.20	rs4654932	4.33E-09	0.1922

HM  =  HapMap, 1 KG  = 1000 Genomes, Uni P*  =  *P*-value derived from univariable analysis after applying genomic control correction. Multi P*  =  *P*-value derived from multivariable analysis after applying genomic control correction.

### Replication and validation

**Figure 5:**
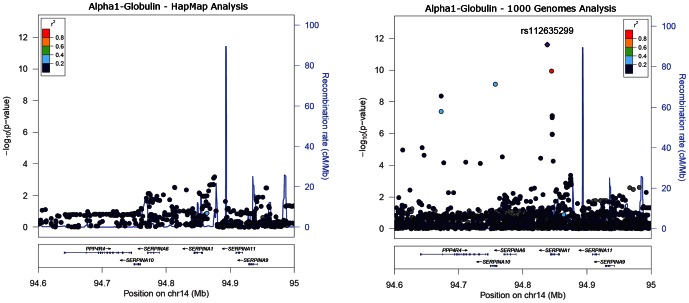
Locus zoom plots of the associations between HapMap and 1000 genomes imputed variants and alpha-1 globulin.

For three of the putative novel associations we attempted to replicate associations in additional data. We used 476 and 529 individuals from the BLSA study to test associations with TNF-alpha and vitamin B12, respectively, and 232, 1554 and 406 individuals from the TwinsUK study to test associations with TNF-alpha, vitamin B12 and Cl^−^, respectively. We did not find any evidence of association. However, one of the putative novel variants does represent a real association. On closer inspection of the literature and comparison of linkage disequilibrium statistics we showed that the SNP rs112635299 represents the known signal between an amino acid changing variant in the *SERPINA* gene and alpha-1-globulin [14]. This signal is present in our data at 0.007 MAF, is a strong risk factor for the lung disease emphysema and was missed by imputation of HapMap genotypes (the SNP identified is not present in the HapMap reference panel, best *P*-value in the region 2×10^−4^) (**Table 7** and **Figure 5**).

**Table 7:** Replication data from three biomarker phenotypes available in the BLSA and or TwinsUK studies.

Trait	1000 G Index SNP	MAF	*P*-value	Beta	BLSA Replication *P*	TwinsUK Replication *P*
TNF-a	rs188141385	0.004	4.20E-10	2.1	0.38	0.56
Cl-	rs2371549	0.005	5.40E-09	2.06	–	0.9
Vitamin B12	rs146226203	0.01	3.00E-08	1.38	0.35	0.41

Beta  =  standard deviations from the double inverse-normalised phenotype values.

## Discussion

Our results show that imputation of genotype data from the sequenced based 1000 Genomes reference panel successfully captures many more variants than imputation of genotype data from the microarray based HapMap reference panel. As expected, this increase in variants captured was much greater for low frequency and rare SNPs compared to common SNPs.

The main purpose of our study was to assess how imputation of missing genotypes from the sequenced based 1000 Genomes reference panel improves our ability to detect genotype-phenotype associations compared to the microarray-genotype based HapMap reference panel. Our results provide two main conclusions. First, that imputation of 1000 Genomes genotype data marginally improves the strength of association signals at known loci. Of 20 associations detected at *P*<5×10^−8^ in both analyses (17 of which represent well replicated signals in the NHGRI catalogue), six were captured by the same index SNP, five were more strongly associated in 1000 Genomes imputed data and one was nominally more strongly associated in HapMap imputed data (based on multivariable statistics **Table 6**). This result was not surprising given that most known associations involve common variants that are likely to be well imputed by both reference datasets. Nevertheless, it is possible that associations undetected by HapMap can be appreciably stronger when using 1000 Genomes imputation, as previously reported in *cis*-eQTL data [15]. We also detected previously unreported associations between known signals and traits. The signals between variants near *SUGP1/TM6SF2* and vitamin E and *APOB* and oxidised LDL were both previously reported with triglycerides and LDL cholesterol. Vitamin E is a lipid-soluble vitamin and associations between lipid variants and vitamin levels have been described before [16].

The second main conclusion is that imputation from 1000 Genomes data can detect associations between low frequency variants and phenotypes that were previously missed by HapMap based imputation approaches. The association between rs112635299 and alpha-1 globulin (%) near the *SERPINA* gene represents the same signal as that detected between rs28929474 (MAF = 0.007; *r* ^2^ = 0.88) and alpha1-antitrypsin that predisposes to emphysema [14]. Although previously known, this association provides important proof of principle that 1000 Genomes imputation can detect novel, low frequency-large effect associations, and potentially save costs of sequencing within studies. One additional association reached our stricter cut-off of *P*<5×10^−11^ in 1000 Genomes based imputation that was not previously reported. Although we have no replication data, the signal between rs113341849 near the *CCR1* and *CCR2* genes and Macrophage inflammatory protein-1b is likely to be real given that these genes encode the receptors to which the MIP1b proteins bind.

A third point emerging from our data is that the selection of appropriate statistical thresholds is important when testing more than one trait and many more variants. Of three putative low-frequency-large effect signals we were able to follow up in additional samples none showed evidence of replication. We note that 7 of the 8 putative low frequency-large effect novel associations identified in the 1000 Genomes based data did not reach our stricter cut-off of *P*<5×10^−11^, and suspect that most represent false positives due to multiple testing.

The main strength of our study was that we tested a large number of circulating biomarkers. Of these phenotypes, 20 had known signals for the same trait, detectable at genome-wide significance in our single study of 1210 individuals. This large number of phenotypes allowed us to test the extent to which 1000 Genomes imputation enhances known association signals. One limitation to our study is the relatively small sample size. Imputation from the 1000 Genomes reference panel may be more beneficial for strengthening associations at known loci in larger sample sizes. Nevertheless, although we only used 1210 individuals, 7 of the known signals were associated at *P*<5×10^−12^ and 3 at *P*<x10^−15^, and therefore provided similarly strong association signals to those detected in larger sample sizes for disease traits. A second limitation to our study is that we did not use the very latest version of 1000 genomes and we only tested one method of imputation (MACH). Our study used the version I panel that includes 1094 individuals and 27,713,623 non-singleton SNPs whereas the latest version includes 1092 individuals and 28,681,763 non-singleton SNPs plus indels. Our data therefore provide a lower bound on the gains to be made from 1000 genomes imputation, although a comparison of association statistics for one biomarker and one chromosome with a known strong signal (chromosome 14 and alpha 1 globulin; **Figure S3**) suggests that any improvement from version I to version III will not be large.

In conclusion, imputation of missing genotypes from sequenced based 1000 Genomes reference data can detect novel genotype-phenotype associations but may not appreciably enhance known signals.

## Methods

### Samples

We used DNA from 1,210 individuals from the population based InCHIANTI study; a study of aging from the Chianti region in Tuscany, Italy [7], [8] (**Table 1**). All summary statistics based association data is available on request from the authors or from the InCHIANTI study door website at http://www.inchiantistudy.net/.

### Phenotypes

We selected 93 circulating factors measured in the InCHIANTI study (**Table S1**). These circulating factors included lipids, proteins, (including binding proteins and hormones), vitamins and ions.

### Genotyping and Imputation

Genome-wide genotyping was performed using the Illumina Infinium HumanHap550 genotyping chip. Standard quality control procedures were used to exclude individuals with discordant sex and call rates less than 98% and filter out SNPs with MAF <1%, Hardy Weinberg *P*<1×10^−4^ and a call rate <99% [8]. There were no ethnic outliers based on a principle components analysis [8]. This resulted in data from 495,343 directly genotyped SNPs in 1,210 individuals. These 495,343 SNPs were used as the scaffold for HapMap and 1000 Genomes based imputation.

For HapMap based imputation we used MACH 1.0.16 to impute missing genotypes not captured by the Illumina chip. We used the HapMap r22 build-36 reference panel (CEU) and imputed 2,543,887 SNPs. The reference panel comprised of 120 haplotypes from 60 parents of 30 Caucasian trios. Of these 2,543,887 SNPs, we excluded 50,205 that were imputed with an *r* ^2^_imp_<0.3 (a measure of imputation quality). This left 2,493,682 SNPs for association analyses.

To perform 1000 Genomes based imputation we estimated phase of contiguous variants in the InCHIANTI subjects using the haplotypes calculated using data from the 1000 Genomes Project consisting of 1,094 individuals and 2,188 haplotypes and the program MACH 1.0.16. We then imputed the variants in the build-37 November 2010 release of 1000 Genomes (Phase 1-α interim) into the phased haplotypes using MINIMAC. This resulted in 37,426,733 imputed SNPs. Of these 37,426,733 SNPs, we excluded 26,547,618 that were imputed with an *r* ^2^_imp_<0.5. We used a more stringent cut-off for 1000 Genomes imputation as recommended at time of analysis. This left 10,879,115 SNPs for association analyses. We used a multi-ethnic reference panel that included 381 Europeans (including 98 Tuscans), 181 Americans, 246 Africans and 286 Asians in an attempt to capture variants that may be rare in Europeans but more common on haplotypes from different ethnic backgrounds.

### Association analysis

Each phenotype measured at baseline was inverse normalised before age and sex adjusted residuals were generated. A second inverse-normalisation was subsequently applied to the residuals. We ran genome-wide association analysis using MACH2QTL for each trait using both HapMap and 1000 Genomes imputed datasets. We performed a univariable test with dosages representing the index HapMap or index 1000 Genomes SNP as independent variables in an additive genetic model and the normalized residuals of the relevant phenotypes as dependent variables in a linear regression analysis.

### Linkage disequilibrium estimates

We estimated linkage disequilibrium within the InCHIANTI data. We used 1000 Genomes imputed dosages (except for two SNPs, where we used HapMap-based dosages because either the SNP was not available in 1000 Genomes imputation reference panel or it failed 1000 Genomes quality control based on the imputation-*r* ^2^) and converted to best-guess genotypes. PLINK was then used to obtain linkage disequilibrium estimates of the SNPs of interest within a given locus.

### Statistical thresholds

We report associations at two statistical thresholds. First, we used a *P*-value of 5×10^−11^ to correct the usual threshold of 5×10^−8^ for the ∼100 traits analysed and the ∼10 fold increase in signals tested (based on 10 million tests arising from 1000 Genomes imputation). This threshold is likely to be conservative given that the 10 million variants are not independent. There is also a Bayesian argument that reasonably presumes that more causal variants exist for more traits and that capturing more variants should result in more true positive signals of association. This argument, combined with the fact that many associations were known means we also report results at the traditional 5×10^−8^.

### Known vs. novel Signals

We downloaded the catalogue of all association signals reaching genome-wide significance (available from the National Human Genome Research Institute (NHGRI), part of National Institutes of Health). We obtained lists of all known SNPs correlated with *r* ^2^>0.2 in Europeans with our index genome wide significant SNPs using the program SNAP (available from the BROAD Institute). We linked this list to phenotype-associated index SNPs using both the HapMap and 1000 Genomes based imputation results.

We classified a *signal* as known if our index SNP or an *r* ^2^>0.8 proxy was associated with the same phenotype or a closely related phenotype (e.g. fatty acids and other lipids) in the NHGRI catalogue (at *P*<5×10^−8^). We classified a *locus* as known if our index SNP or an *r* ^2^>0.2 and *r* ^2^<0.8 proxy was associated with the same phenotype in the NHGRI catalogue (*P*<5×10^−8^).

As the NHGRI catalogue is not 100% complete, we also conducted literature searches using the name of the circulating factor and other key terms including “GWAS” or “Genome wide”.

### Does 1000 Genomes imputation identify stronger associations at loci identified by HapMap based imputation?

For all loci reaching genome-wide significance in the HapMap and 1000 Genomes based analysis we performed additional statistical tests to assess whether or not 1000 Genomes based imputation identifies stronger associations. We performed a multivariable test with dosages representing both the index HapMap and index 1000 Genomes SNP as independent variables (where the two index SNPs were different) in an additive genetic model and the normalized residuals of the relevant phenotypes as dependent variables in a linear regression analysis. We used 1000 Genomes imputed dosages to represent SNPs identified by both HapMap and 1000 Genomes analyses in STATA. Where 1000 Genomes based dosages were not available we extracted the dosage from the HapMap imputation dataset. We compared the strength of association explained between univariable and multivariable SNP analyses.

### Replication and validation

For three of the putative novel associations we attempted to replicate associations in additional data. We used 476 and 529 individuals from the BLSA study [17] to test associations with TNF-alpha and vitamin B12, respectively. In addition, 232, 1554 and 4068 individuals from the TwinsUK study [18] were used to test associations with TNF-alpha, vitamin B12 and chlorine ion levels, respectively.

### Data availability

Genome wide summary statistics from all analyses are available from the authors on request. Please contact corresponding authors.

## Supporting Information

Figure S1 Distribution of SNP minor allele frequencies (MAFs) within imputation *r* ^2^ categories.
(DOC)

Figure S2 Distributions of the inverse normalised residual values of the 6 traits with evidence of low-frequency/large effect variants as captured by 1000 Genomes imputation.
(DOC)

Figure S3 A comparison of P-values when testing Alpha 1 globulin using overlapping SNPs present in both 1000 Genomes Phase 1 versions 1 and 3 on chromosome 14. Correlation coefficient  = 0.931.
(DOC)

Table S1 The 93 circulating factors analysed.
(XLSX)

Table S2 HapMap and 1000 Genomes based SNP counts per minor allele frequency (MAF) and imputation quality metric (*r* ^2^) bin.
(XLSX)
