# Supplementary material for: Imputation of Variants from the 1000 Genomes Project Modestly Improves Known Associations and Can Identify Low-frequency Variant - Phenotype Associations Undetected by HapMap Based Imputation
Source: PLoS One. 2013 May 16;8(5):e64343. doi: 10.1371/journal.pone.0064343 (PMC3655956; doi:10.1371/journal.pone.0064343)
Supplement: Figure S1 — Distribution of SNP minor allele frequencies (MAFs) within imputation r 2 categories. (DOC) [file pone.0064343.s001.doc]

**Figure S1.** Distribution of SNP minor allele frequencies (MAFs) within imputation *r*^2^ categories.


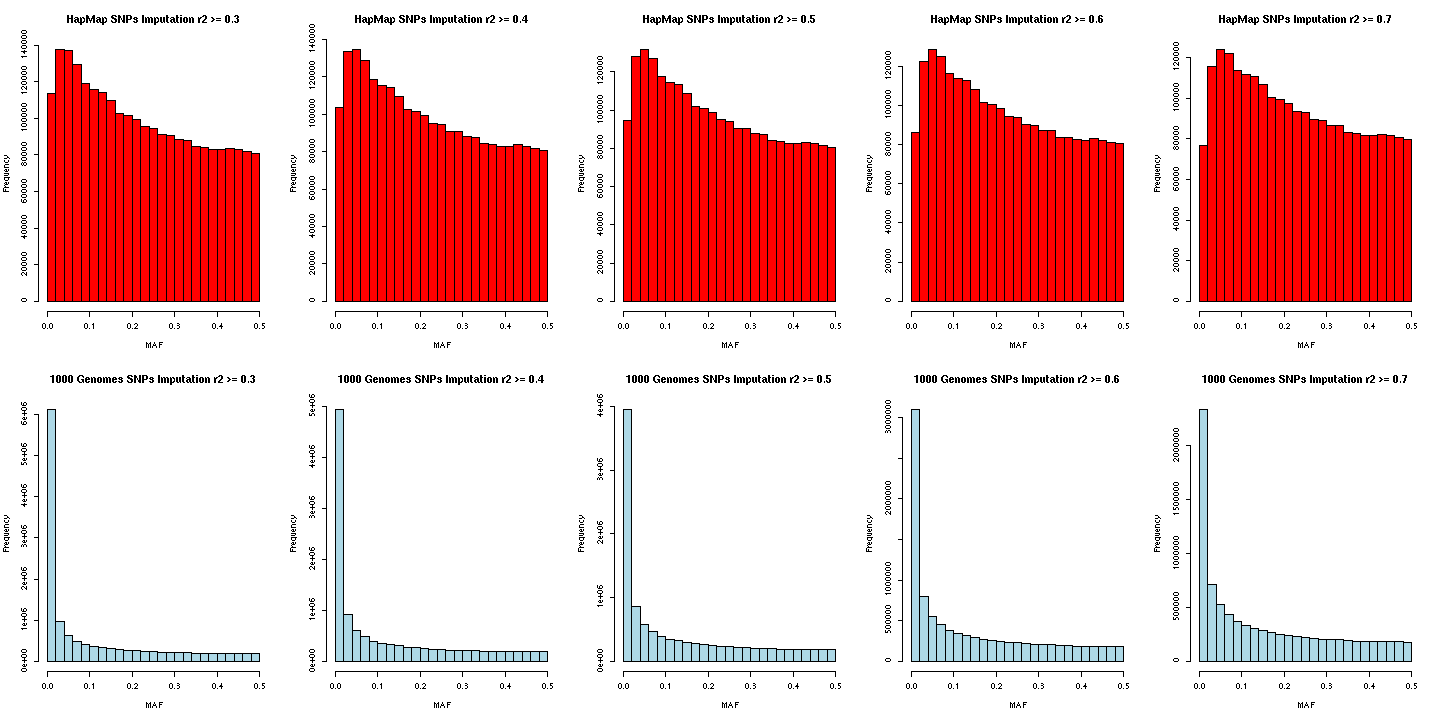


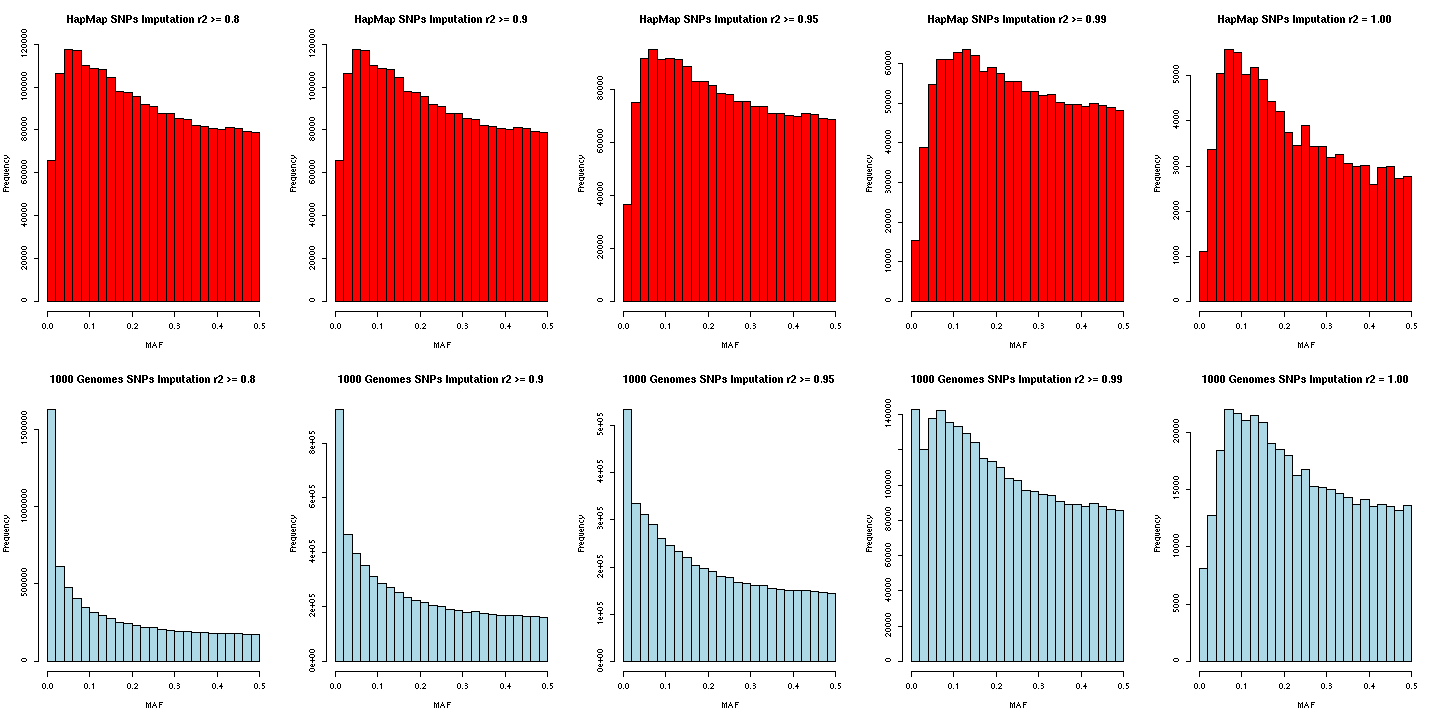
**Figure S1 continued**
