# Supplementary material for: Imputation of Variants from the 1000 Genomes Project Modestly Improves Known Associations and Can Identify Low-frequency Variant - Phenotype Associations Undetected by HapMap Based Imputation
Source: PLoS One. 2013 May 16;8(5):e64343. doi: 10.1371/journal.pone.0064343 (PMC3655956; doi:10.1371/journal.pone.0064343)
Supplement: Figure S2 — Distributions of the inverse normalised residual values of the 6 traits with evidence of low-frequency/large effect variants as captured by 1000 Genomes imputation. (DOC) [file pone.0064343.s002.doc]

**Figure S2.** Distributions of the inverse normalised residual values of the 6 traits with evidence of low-frequency/large effect variants as captured by 1000 Genomes imputation.

**
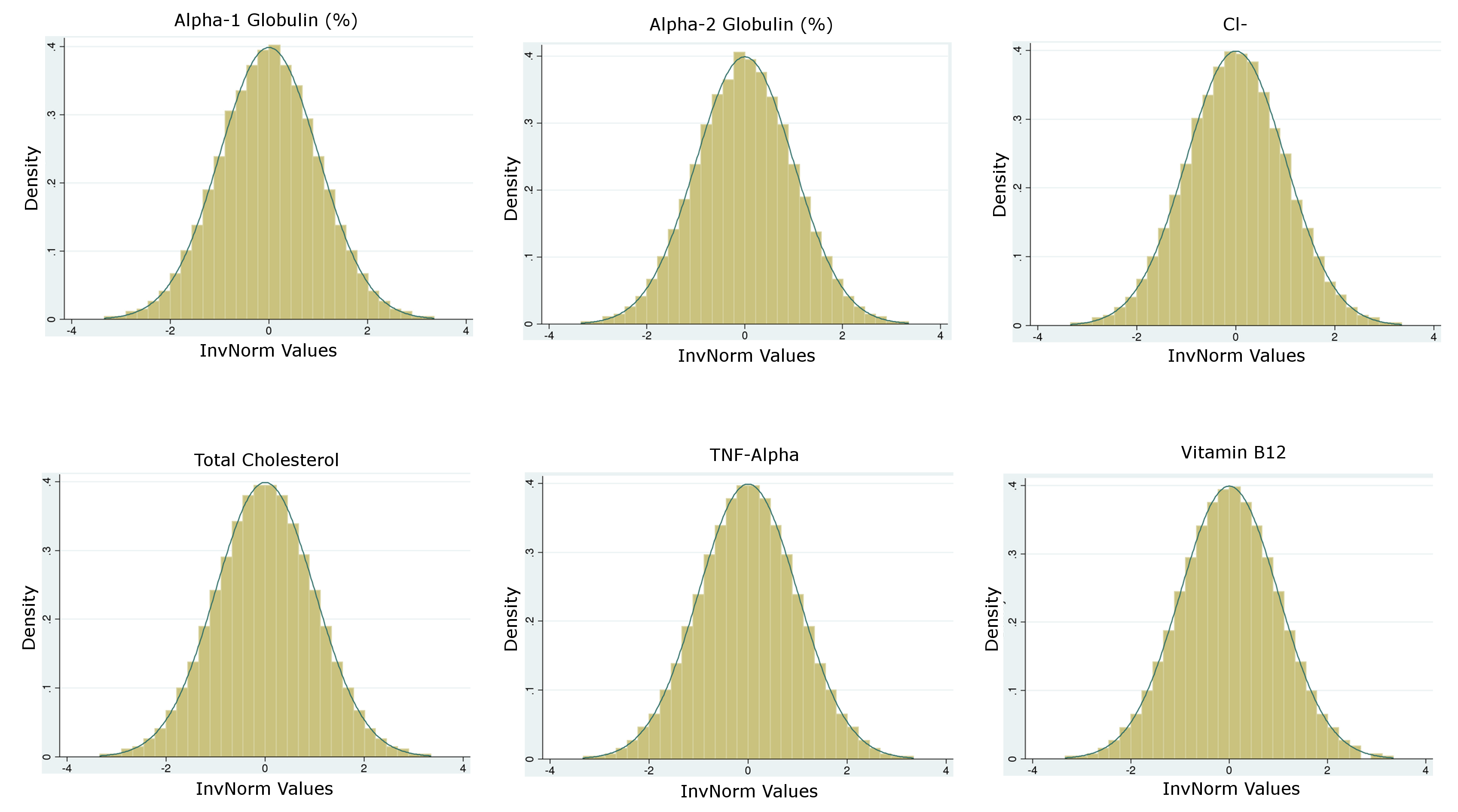
**
