# Supplementary material for: Imputation of Variants from the 1000 Genomes Project Modestly Improves Known Associations and Can Identify Low-frequency Variant - Phenotype Associations Undetected by HapMap Based Imputation
Source: PLoS One. 2013 May 16;8(5):e64343. doi: 10.1371/journal.pone.0064343 (PMC3655956; doi:10.1371/journal.pone.0064343)

**Figure S3**. A comparison of P-values when testing Alpha 1 globulin using overlapping SNPs present in both 1000 Genomes Phase 1 versions 1 and 3 on chromosome 14. Correlation coefficient = 0.931.


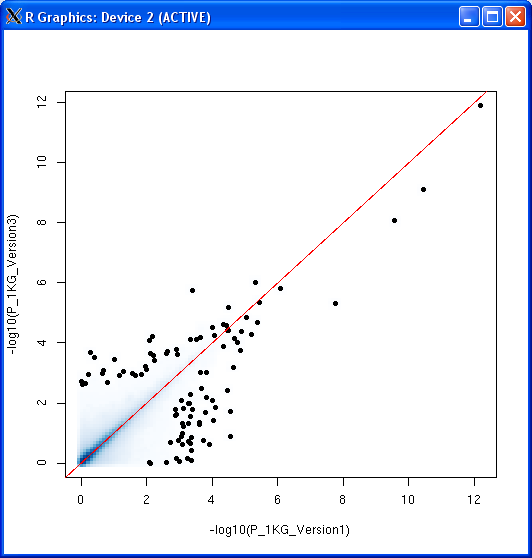

Supplement: Figure S3 — A comparison of P-values when testing Alpha 1 globulin using overlapping SNPs present in both 1000 Genomes Phase 1 versions 1 and 3 on chromosome 14. Correlation coefficient = 0.931. (DOC) [file pone.0064343.s003.doc]
